# Datasets for learning of unknown characteristics of dynamical systems

**DOI:** 10.1038/s41597-023-01978-7

**Published:** 2023-02-07

**Authors:** Agnieszka Szczęsna, Dariusz Augustyn, Katarzyna Harężlak, Henryk Josiński, Adam Świtoński, Paweł Kasprowski

**Affiliations:** 1 Department of Computer Graphics, Vision and Digital Systems, Faculty of Automatic Control, Electronics and Computer Science, Silesian University of Technology, 44-100 Gliwice, Akademicka 16, Poland; 2 Department of Applied Informatics, Faculty of Automatic Control, Electronics and Computer Science, Silesian University of Technology, 44-100 Gliwice, Akademicka 16, Poland

**Keywords:** Biomedical engineering, Scientific data

## Abstract

The ability to uncover characteristics based on empirical measurement is an important step in understanding the underlying system that gives rise to an observed time series. This is especially important for biological signals whose characteristic contributes to the underlying dynamics of the physiological processes. Therefore, by studying such signals, the physiological systems that generate them can be better understood. The datasets presented consist of 33,000 time series of 15 dynamical systems (five chaotic and ten non-chaotic) of the first, second, or third order. Here, the order of a dynamical system means its dimension. The non-chaotic systems were divided into the following classes: periodic, quasi-periodic, and non-periodic. The aim is to propose datasets for machine learning methods, in particular deep learning techniques, to analyze unknown dynamical system characteristics based on obtained time series. In technical validation, three classifications experiments were conducted using two types of neural networks with long short-term memory modules and convolutional layers.

## Background & Summary

Time series obtained from observations collected sequentially over time are very common. The list of areas in which time series are studied is wide. For example, in the economy, we observe closing stock prices, price indices, currency exchange rates, and so forth. In meteorology, we observe temperatures and the amount of precipitation. In agriculture, we can record crop and livestock production, and soil erosion. In the medical sciences, we observe biomedical signals. The purpose of time series analysis is generally twofold: to understand or model the underlying system that gives rise to an observed series and to predict or forecast the future values of a series based on the history of that series^1^. Due to better and more accessible acquisition systems, more and more time series resources are available. An example may be the increasing popularity of wearable devices, which results in the possibility of monitoring biomedical signals in activities of everyday life.

Time series is a series of observations (samples) taken sequentially in time. The size of the datasets is often large which makes the analysis to be computationally expensive. For some of the most challenging real-life applications, a dynamical system model is unknown, which makes the identification of the different dynamical properties impossible. The ability to detect chaos based on the empirical measurement is an significant step in understanding these processes. This is especially important for biological signals whose characteristic contributes to the underlying dynamics of the physiological processes. Therefore, by studying such signals, the physiological systems that generate them can be better understood. This is worthy of attention, that biomedical time series measurements with wearable devices are easily available also during daily life activities and are increasingly used also for the needs of medical diagnosis^2–6^.

In early studies, time series chaotic behaviour was analysed mostly based on the results of time delay reconstructed trajectory, correlation dimension, and largest Lyapunov exponent. Subsequently, with the development of nonlinear time series analysis methods for real-world data, many tools that were previously thought to provide clear evidence of chaotic motion have been found to be sensitive to noise and can produce misleading results. Thus, it is still a quite controversial topic, especially in the field of biomedical signals like PPG (photoplethysmography) as well as ECG (electrocardiogram) and HRV (heart rate variability)^7–9^.

The analysis of the occurrence of chaotic behavior is also important for the biomedical signal representing movement. The first example is exploring eye movement dynamic features in terms of the existence of chaotic nature during fixations^10^ or and for the signal with and without noise removal^11^. Eye movements may be influenced by memory, emotion, or the ability to anticipate and non-chaotic behavior may be an indicator of stress or pathology^12^.

A strong sensitivity to initial conditions, which is the hallmark of chaotic behavior of a dynamical system, is also present in human gait when a locomotor system attenuates the effects of infinitesimally small disruptions that occur naturally in consecutive cycles of gait due to outer factors (e.g., irregularities in the texture of the ground) or inner factors (e.g., presence of noise in neuro-muscular system). This ability of a human locomotor system is called local dynamic stability (LDS)^13^. Several studies (e.g.^14–16^) indicate that LDS is associated with the fall risk. Lack or even weakening of locomotion stability may lead to a serious fall, which is: (i) especially dangerous in consequences in case of older people, (ii) justifies carrying out the research concerning the methods of gait stability estimation, e.g., based on the largest Lyapunov exponent^17^.

The latest approach to signal analysis uses machine learning or deep learning techniques^18^. Such methods allow the knowledge gained from the training set to be generalized and applied to the analysis of the test dataset. Boullé *et al*.^19^ classified univariate time series’ by a deep neural network based on the presence of chaotic behavior. A narrow group of systems was considered in two cases. For discrete dynamic systems, the main goal was to find a neural network that is able to learn the features characterising chaotic signals of the logistic map and generalise on signals generated by the sine-circle map. Chosen systems exhibit periodic or chaotic behaviour depending on the value of the parameter. For continuous dynamical systems the aim was to determine whether a neural network trained on a low dimensional dynamical system is able to generalise and classify univariate time series generated by a higher dimensional dynamical system based on the Lorenz system and Kuramoto–Sivashinsky. The study suggests that deep learning techniques can be used to classify time series obtained by real-life applications into chaotic or non-chaotic.

When examining dynamic systems, it is an important process to identify the signals obtained from the systems or to determine which system they belong to. The results obtained in the study^20^ show the possibility of classifying signals with chaotic characters and associating them with a mathematical model. The training and testing were performed only on three chaotic systems - Lorenz, Chen, and Rössler.

In study^21^, the graphic images of time series of different chaotic systems were classified with deep learning methods. For the classification, a dataset containing images of the time series of Chen and Rössler chaotic systems for different parameter values, initial conditions, step size, and time length were generated. Then, high accuracy classifications were performed with transfer learning methods. The used transfer learning methods are SqueezeNet, VGG-19, AlexNet, ResNet-50, ResNet-101, DenseNet-201, ShuffleNet, and GoogLeNet. According to the problem, classification accuracy is varying between 89% and 99.7%. Thus, this study shows that identifying a chaotic system from its graphic image of time series is possible.

Machine learning methods are also used to predict time signal metrics from empirical data without any assumption on the underlying dynamics. The method can estimate the largest Lyapunov exponent from noisy data, based on training deep learning models on synthetically generated trajectories^22^. The deep learning techniques were trained with different indexing methods based on chaos indicators, like Fast Lyapunov Indicators and the frequency map analysis^23^. The goal was to classify types of motion (series corresponds to a chaotic, librational, or rotational motion) by observing samples taken from time series coming from simple Hamiltonian systems.

A deep hybrid neural network based on a convolutional neural network (CNN), gated recurrent unit (GRU) network, and attention mechanism was used to predict chaotic time series^24^. The Lorenz chaotic time series, monthly mean total sunspot datasets, and the actual coal-mine gas concentration datasets were used to verify the prediction accuracy of the proposed prediction model.

In study^25^, the deep neural network was used to classify biomedical PPG signals. The following classes were defined: periodic, quasi-periodic, non-periodic, chaotic, or random dynamics. Unfortunately, the dataset used to train the deep neural network has only one system for each class.

The presented dataset has already been used in the classification of PPG (photoplethysmographic) signals with the use of features determined on the basis of wavelet scattering transform^26^. The presented research results indicate the need to prepare an appropriate training dataset for the classification of time series characteristics.

The aim is to propose a dataset for machine learning methods, in particular deep learning techniques, to analyze unknown dynamical system characteristics based on obtained time series. The dataset contains signals determined on the basis of systems with known characteristics. The main division is the signals generated by chaotic, and non-chaotic systems. Non-chaotic ones were divided into the following classes: periodic, quasi-periodic, and non-periodic. The specific noise can be easily added to signals by building a model that takes into account the measurement noise. This is especially important for the analysis of biomedical signals. The model trained based on the presented dataset can be used for the classification of signals, determining the parameters of the underlining system, time series forecasting, replenishment of missing samples, or generating signals with specific properties. According to our knowledge, no such dataset has been made available, yet.

## Methods

Having in mind a diverse representations of the analyzed signals, data for training and testing sets were generated using models of 15 dynamical systems (five chaotic and ten non-chaotic) of the first, second, or third order. Here, the order of a dynamical system means its dimension. Tables 1–4 contain detailed characteristics of the models: name and type (*Class*), number of state variables (*Dim*), state equations, initial conditions of state variables and final value of the independent variable *t* (*T*_*max*_), model parameters, brief description, sample graph of state variables over time, and phase portrait. Assuming the following definitions: *m* – number of models (*m* = 15), *v* – number of initial vectors for each model (*v* = 1,000), *n*_*j*_ for *j* = 1...m number of state variables or signals for the *j*-th model (*n*_*j*_∈{1, 2, 3}), *s* – number of samples for each initial vector (i.e., the length of a time series; *s* = 1,000), the total number of time series is ${\Sigma }_{j=1}^{m}{n}_{j}\cdot v=33\cdot 1,000=33,000$. As a consequence, a set of 1,000 test files was created for each system, each containing 1,000 samples. Augmentation was applied by randomizing the initial conditions, represented by vector that each element was obtained by multiplying [2·*rand*()−1] by the corresponding element of vector *x*_0_ (see: Tables 1–4), where *rand*() generates pseudorandom numbers that are uniformly distributed in the interval (0, 1).

**Table 1:** Detailed characteristics of chaotic signals.

Symbol	Properties				Sample signal	Sample phase portrait
CHA_1^35^ A.4.5	Name	*Ueda oscillator*			Fig. 1	Fig. 2
	Class	chaotic	Dim	2		
	Params	*b* = 0.05, *A* = 7.5, Ω = 1				
	Init. cond.	*x*_0_ = [2.5, 0], *T*_*max*_ = 100				
	State eqn.	${\mathop{x}\limits^{.}}_{1}={x}_{2}$				
		$${\mathop{x}\limits^{.}}_{2}=-{x}_{1}^{3}-b{x}_{2}+Asin(\Omega t)$$				
	Descr.	Driven dissipative flow				
CHA_2^36^ A.5.1	Name	*Lorenz attractor*			Fig. 3	Fig. 4
	Class	chaotic	Dim	3		
	Params	$\sigma =10,\beta =8/3,\rho =28$				
	Init. cond.	*x*_0_ = [10, 200, 10], *T*_*max*_ = 100				
	State eqn.	${\mathop{x}\limits^{.}}_{1}=-\sigma {x}_{1}+\sigma {x}_{2}$				
		$${\mathop{x}\limits^{.}}_{2}=\rho {x}_{1}-{x}_{2}-{x}_{1}{x}_{3}$$				
		$${\mathop{x}\limits^{.}}_{3}=-\beta {x}_{3}+{x}_{1}{x}_{2}$$				
	Descr.	Autonomous dissipative flow				
CHA_3^37^ A.5.2	Name	*Rössler attractor*			Fig. 5	Fig. 6
	Class	chaotic	Dim	3		
	Params	$a=0.2,b=0.2,c=5.7$				
	Init. cond.	*x*_0_ = [−9, 0, 0], *T*_*max*_ = 300				
	State eqn.	${\mathop{x}\limits^{.}}_{1}=-{x}_{2}-{x}_{3}$				
		$${\mathop{x}\limits^{.}}_{2}={x}_{1}+a{x}_{2}$$				
		$${\mathop{x}\limits^{.}}_{3}=b+{x}_{3}({x}_{1}-c)$$				
	Descr.	Autonomous dissipative flow				
CHA_4^38^ A.5.13	Name	*Halvorsen attractor*			Fig. 7	Fig. 8
	Class	chaotic	Dim	3		
	Params	$a=1.27$				
	Init. cond.	*x*_0_ = [−5, 0, 0], *T*_*max*_ = 50				
	State eqn.	${\mathop{x}\limits^{.}}_{1}=-a{x}_{1}-4{x}_{2}-4{x}_{3}-{x}_{2}^{2}$				
		$${\mathop{x}\limits^{.}}_{2}=-4{x}_{1}-a{x}_{2}-4{x}_{3}-{x}_{3}^{2}$$				
		$${\mathop{x}\limits^{.}}_{3}=-4{x}_{1}-4{x}_{2}-a{x}_{3}-{x}_{1}^{2}$$				
	Descr.	Autonomous dissipative flow (cyclically symmetric attractor)				
CHA_5^39^ A.5.15	Name	*Rucklidge attractor*			Fig. 9	Fig. 10
	Class	chaotic	Dim	3		
	Params	*k* = 2, *λ* = 6.7				
	Init. cond.	*x*_0_ = [1, 0, 4.5], *T*_*max*_ = 150				
	State eqn.	${\mathop{x}\limits^{.}}_{1}=-k{x}_{1}+\lambda {x}_{2}-{x}_{2}{x}_{3}$				
		$${\mathop{x}\limits^{.}}_{2}={x}_{1}$$				
		$${\mathop{x}\limits^{.}}_{3}=-{x}_{3}+{x}_{2}^{2}$$				
	Descr.	Autonomous dissipative flow

**Table 2:** Detailed characteristics of the non-chaotic, periodic signals.

Symbol	Properties				Sample signal	Sample phase portrait
OSC_1^40^	Name	*Undamped oscillator 1*			Fig. 11	Fig. 12
	Class	periodic	Dim	2		
	Params	*a* = 0.5, *b* = −0.5				
	Init. cond.	*x*_0_ = [1, 1], *T*_*max*_ = 100				
	State eqn.	${\mathop{x}\limits^{.}}_{1}=a{x}_{2}$				
		$${\mathop{x}\limits^{.}}_{2}=b{x}_{1}$$				
	Descr.	Linear continuous dynamical system – slow oscillation with constant amplitude				
OSC_2^41^	Name	*Undamped oscillator 2*			Fig. 13	Fig. 14
	Class	periodic	Dim	2		
	Params	*a* = 0.5, *b* = −0.5				
	Init. cond.	*x*_0_ = [0.9, −0.9], *T*_*max*_ = 250				
	State eqn.	${\mathop{x}\limits^{.}}_{1}=a{x}_{2}$				
		$${\mathop{x}\limits^{.}}_{2}=b{x}_{1}$$				
	Descr.	Linear continuous dynamical system – fast oscillation with constant amplitude				
DOSC_1^42^	Name	*Damped oscillator 1*			Fig. 15	Fig. 16
	Class	periodic	Dim	2		
	Init. cond.	*x*_0_ = [1, 1], *T*_*max*_ = 100				
	State eqn.	${\mathop{x}\limits^{.}}_{1}={x}_{2}$				
		$${\mathop{x}\limits^{.}}_{2}=-0.2{x}_{1}-0.08{x}_{2}+0.01$$				
	Descr.	Linear continuous dynamical system – fast oscillations with decreasing amplitude				
DOSC_2^43^	Name	*Damped oscillator 2*			Fig. 17	Fig. 18
	Class	periodic	Dim	2		
	Init. cond.	*x*_0_ = [0.4, 0.3], *T*_*max*_ = 100				
	State eqn.	${\mathop{x}\limits^{.}}_{1}={x}_{2}$				
		$${\mathop{x}\limits^{.}}_{2}=-0.04{x}_{1}-0.016{x}_{2}+0.01$$				
	Descr.	Linear continuous dynamical system – slow oscillations with decreasing amplitude				
IOSC^44^	Name	*Rising oscillator*			Fig. 19	Fig. 20
	Class	periodic	Dim	2		
	Init. cond.	*x*_0_ = [0.1, 0.1], *T*_*max*_ = 100				
	State eqn.	${\mathop{x}\limits^{.}}_{1}={x}_{2}$				
		$${\mathop{x}\limits^{.}}_{2}=-0.2{x}_{1}+0.08{x}_{2}+0.01$$				
	Descr.	Linear continuous dynamical system – oscillations with growing amplitude

**Table 3:** Detailed characteristics of the non-chaotic, quasi-periodic signals.

Symbol	Properties				Sample signal	Sample phase portrait
QPS_1^45^	Name	*Quasi-periodic 1*			Fig. 21	Fig. 22
	Class	quasi-periodic	Dim	1		
	Params	*ω*_1_ = *π*				
	Init. cond.	*x*_0_ = 2*π*, *T*_*max*_ = 1000				
	State eqn.	$x=cos({\omega }_{1}t/50+{x}_{0})+cos(t/50+{x}_{0})$				
	Descr.	Irrational *ω* ratio: *ω*_1_/*ω*_2_ = *π*				
QPS_2^46^	Name	*Quasi-periodic 2*			Fig. 23	Fig. 24
	Class	quasi-periodic	Dim	1		
	Params	${\omega }_{1}=(1+\sqrt{5})/2$				
	Init. cond.	*x*_0_ = 2*π*, *T*_*max*_ = 1000				
	State eqn.	$x=sin({\omega }_{1}t/15+{x}_{0})+sin(t/15+{x}_{0})$				
	Descr.	Irrational *ω* golden ratio: ${\omega }_{1}/{\omega }_{2}=\varphi =(1+\sqrt{5})/2$				
QPS_3^47^	Name	*Quasi-periodic 3*			Fig. 25	Fig. 26
	Class	quasi-periodic	Dim	1		
	Params	*ω*_1_ = *e* = 2.71828…				
	Init. cond.	*x*_0_ = 2*π*, *T*_*max*_ = 1000				
	State eqn.	$x=sin({\omega }_{1}t/15+{x}_{0})+cos(t/15+{x}_{0})$				
	Descr.	Irrational *ω* ratio: *ω*_1_/*ω*_2_ = *e* = 2.71828…

**Table 4:** Detailed characteristics of the non-chaotic, non-periodic signals.

Symbol	Properties				Sample signal	Sample phase portrait
DS_1^48^	Name	*Damped system 1*			Fig. 27	Fig. 28
	Class	non-periodic	Dim	3		
	Init. cond.	*x*_0_ = [1.8, 2.3, 3], *T*_*max*_ = 100				
	State eqn.	${\mathop{x}\limits^{.}}_{1}=-0.01{x}_{1}+0.01{x}_{2}$				
		$${\mathop{x}\limits^{.}}_{2}=-0.001{x}_{1}-0.01{x}_{2}$$				
		$${\mathop{x}\limits^{.}}_{3}=\quad 0.05{x}_{1}-0.03{x}_{3}$$				
	Descr.	Linear continuous dynamical system – slow fading signals				
DS_2^49^	Name	*Damped system 2*			Fig. 29	Fig. 30
	Class	non-periodic	Dim	3		
	Init. cond.	*x*_0_ = [3, −1, 2], *T*_*max*_ = 100				
	State eqn.	${\mathop{x}\limits^{.}}_{1}=-0.05{x}_{1}+0.01{x}_{2}$				
		$${\mathop{x}\limits^{.}}_{2}=-0.001{x}_{1}-0.01{x}_{2}$$				
		$${\mathop{x}\limits^{.}}_{3}=\quad 0.05{x}_{1}-0.08{x}_{3}$$				
	Descr.	Linear continuous dynamical system – fast fading signals				

In the case of models described by a system of first-order differential state equations, the MATLAB *obe*45 method was used to compute values of state variables over a range of values of the independent variable *t* from 0 to *T*_*max*_ with adjustment of the integration step size. Finally, interpolation at equidistant time points was performed.

The chaotic systems (Table 1) are represented by driven or autonomous dissipative flows. Previously described in^27^ as A.4.5, A.5.1, A.5.2, A.5.13, and A.5.15, we denote here these flows as CHA_1, CHA_2, CHA_3, CHA_4, and CHA_5, respectively. The non-chaotic systems were divided into the following classes: i) periodic (Table 2), including the OSC_1, OSC_2, DOSC_1, DOSC_2, and IOSC systems; ii) quasi-periodic (Table 3), including QPS_1, QPS_2, and QPS_3; and iii) non-periodic (Table 4), including DS_1 and DS_2. Definitions of proposed quasi-periodic signals are presented in Table 3. The considered quasi-periodic systems are described by the following general function $x=f(t)={A}_{1}\cdot sin\left({\omega }_{1}\cdot t+{\varphi }_{1}\right)+{A}_{2}\cdot sin\left({\omega }_{2}\cdot t+{\varphi }_{2}\right)$, where the ratio *ω*_1/_*ω*_2_ is irrational. The directly defined signals were used for quasi-periodic models because of clear form of such definition (irrational ratio of angular frequencies of oscillated addends) that seems to be very intuitive for a reader and consistent, comparing to the model given by ODEs. It is rather not possible to use such approach (i.e., to give the explicit form of formula which describes a signal respect to time) for chaotic models.

Generally, quasi-periodic signals are typical for turbulence studies in fluid dynamics. Some false seeming “repetitive structure” property of a quasi-periodic signal results only from a low accuracy of its representation generated during a numerical simulation. We can define a signal as a quasi-periodic if it generates a torus in a phase space with a sufficiently high dimension. Here the torus denotes a surface without the enclosed volume^28^. Thus, each signal QPS_1,…, QPS_3 generates a portrait which is a torus in a 3D space. Figures in the last column of Table 3 show the phase portraits for QPS_1… QPS_3 in phase spaces where the axes are: signal, approximation of the first-order derivative of the signal, and approximation of the second-order derivative of the signal. The values for the figures were calculated using the MATLAB code^29^ that is based on Takens theorem^30^.

There is a class of dynamical systems, described by an *n*th order ordinary differential equation with *n* > 3, referred to as hyperjerk systems, which can be regarded as general and prototypical examples of complex dynamical systems in a high-dimensional phase space^31^. For this reason, it is worth considering chaotic hyperjerk systems or, more generally, chaotic systems with *n* > 3 in future research. It’s worthwhile to mention that such systems can be constructed using a general method based on non-linearity represented by the hyperbolic sine function^32^.

## Data Records

The description of the prepared datasets is available via the following website^33^. It includes the following elements: definitions of the dynamical models (given by state equations) or signals (given by formulas of signal values with respect to time) used to generate datasets, sample time plots, and phase portraits for each type of signal. The datasets themselves are also available through the aforementioned website. Detailed description of models characteristics is also in Tables 1–4.

Direct references for downloading all data files are enabled by the figshare data repository project^34^. Each zip archive for each model contains 1,000 files (in CSV format). They were generated in the MATLAB environment by running 1,000 simulations with random initial conditions for a given dynamical model. Each CSV file contains 1,000 samples (i.e., 1,000 rows). Each column of a file includes values of one state variable, so the number of columns is the state-space dimensionality of a dynamical model. List of model symbols and references to data files enabled by the figshare project^34^: CHA_1^35^, CHA_2^36^, CHA_3^37^, CHA_4^38^, CHA_5^39^, OSC_1^40^, OSC_2^41^, DOSC_1^42^, DOSC_2^43^, IOSC^44^, QPS_1^45^, QPS_2^46^, QPS_3^47^, DS_1^48^, DS_2^49^.

## Technical Validation

Several experiments were conducted to demonstrate the usability of the generated signals and establish a baseline for future studies. Their purpose was to provide evidence that the elaborated dataset may be used for defining models able to distinguish signals with chaotic characteristics from non-chaotic behaviour. The one-dimensional courses were considered; therefore, each state variable was used as a single signal for multidimensional dynamical models. Additionally, the analysis was conducted by taking different signal scopes into account. Each sequence of initial vectors including *s* samples (*s* = 1,000) was divided into subsets consisting of Y ∈{50, 100, 200} samples. These signal segments constituted the feature vectors for feeding two types of neural networks which are based on long short-term memory (LSTM) modules and convolutional layers.

LSTMs are types of recurrent neural networks with feedback connections, capable to process time series data with different duration and resistant to the problem of vanishing gradient. Their basic components are LSTM units with recurrent structure. They have *forget*, *input* and *output* gates with assigned weight and bias coefficients. Every unit has its own current and hidden states. Forget mechanism based on the hidden state and input data decides how strongly the current state is modified. LSTM layers and networks are formed by the described units. During the training stage, all weight and bias coefficients are determined. There are different strategies used in learning. By default, the output of the networks - the actual class value - is expected to be properly predicted at the end of the time series. It means a complete pass of the time series is performed before parameter update and prediction. However, there are also variants in which class is specified and determined separately for every time instant. They are mainly used in segmentation challenges.

Convolutional layers are extremely efficient in the recognition of image data and they are simplified variants of fully connected ones. They convolve the input, which means the sum of products in local neighborhoods of input data and kernels are calculated. It makes the processing to be invariant to translation. Every layer is specified by the number of kernels, their dimensionality, and resolution. The kernel coefficients are determined during the training stage. The networks containing such layers are called convolutional neural networks (CNN).

For the typical CNN, the results of convolutions are flattened and sent to the classic dense layers where every neuron is connected with every output from the previous layer. It results in the limitation that the dimensionality of input samples must be fixed for the trained model and must be the same for each processed sample. Thus, only time series with the same length can be classified, which is satisfied in our experiments. We have univariate sequences, represented by the first-order tensors. Thus, one-dimensional convolutions operating in a time domain are applied. To suppress overfitting phenomena and for faster learning, two regularization techniques were used. It was the dropout mechanism that ignores during the training stage, the specified percentage of randomly selected outputs of a layer in every iteration. The second one was batch normalization, standardizing input layer values for every batch.

All experiments were conducted in Python language with the usage of TensorFlow platform version 2.7.0. For the assessment of the developed models, the accuracy metric was utilized. Additionally,

- true positives (TP) - samples correctly classified as positive,
- false negatives (FN) - samples incorrectly classified as negative,
- false positives (FP) - samples incorrectly classified as positive,
- true negatives (TN) - samples correctly classified as negative),

for each experiment, each model, and each segment size were presented. In all binary classifications, the signals with chaotic behaviour (chaos class) were denoted as a positive class and non-chaos as negative.

### Experiment 1

The first experiment aimed to ascertain the influence of a time series length on classification efficiency. Therefore, whole signals were split into segments containing the aforementioned numbers of samples. This division was realized for each segment size independently (Fig. 31). In this study’s stage, the attention was focused on signals featuring chaotic behaviour as one class, and periodic and non-periodic signals as another class. From 15 available signals, the representative subset consisting of seven elements was utilized. In the first group, the Lorenz (CHA_2) and Rössler attractors (CHA_3), and Ueda oscillator (CHA_1) were chosen. From the second group, the Undamped oscillator 1 (OSC_1), Damped oscillator 02 (DOSC_2), Rising oscillator (IOSC), and Damped system 1 (DS_1) were selected. They were utilized to define counterbalanced train and test sets with two class labels. The size of sets constructed in such a way depended on the signal’s part utilized for the classification, which is shown in Table 5, columns 2 and 3.

**Fig. 1:**
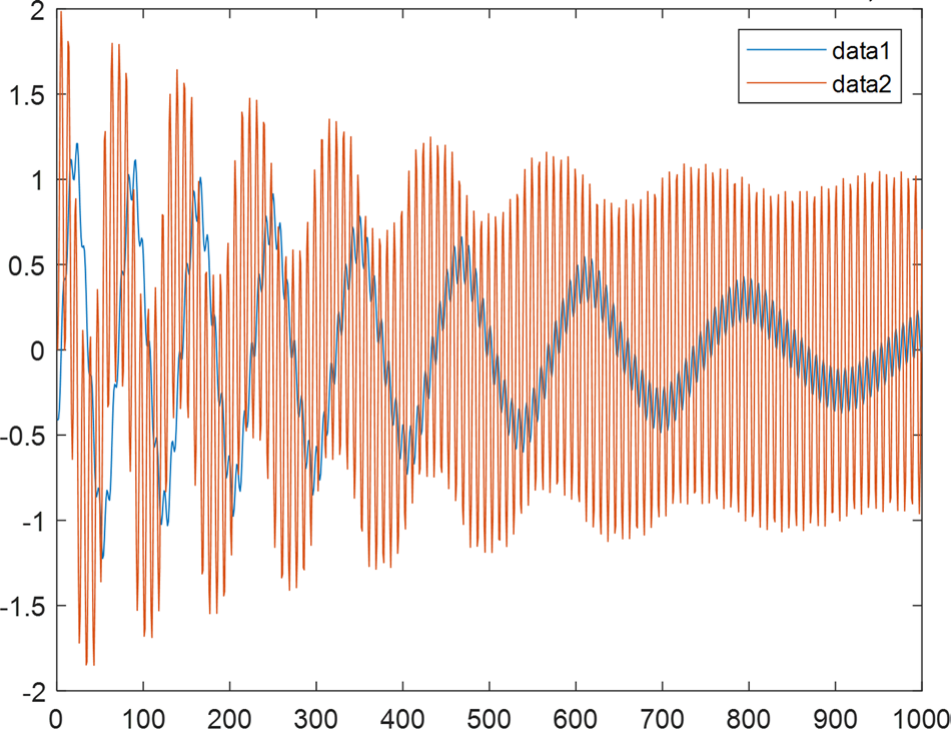
CHA_1 - sample signal.

**Fig. 2:**
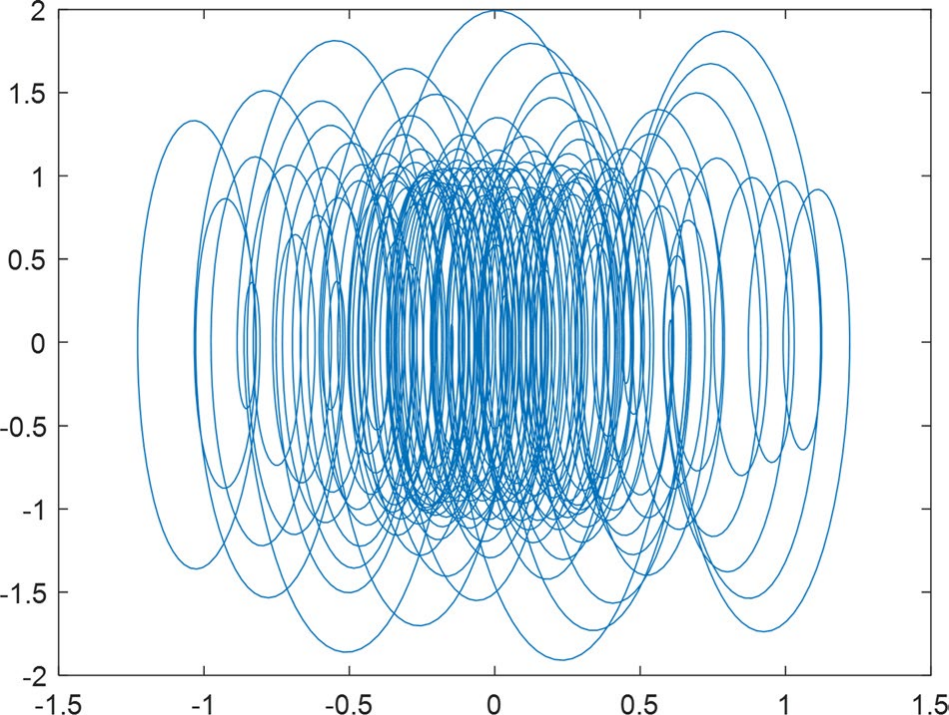
CHA_1 - phase portrait.

**Fig. 3:**
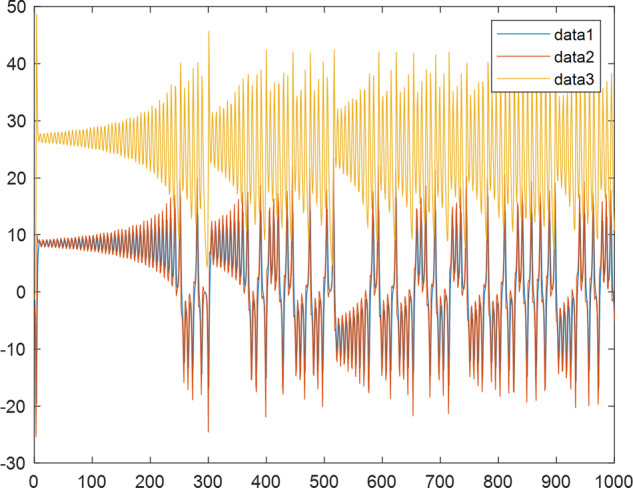
CHA_2 - sample signal.

**Fig. 4:**
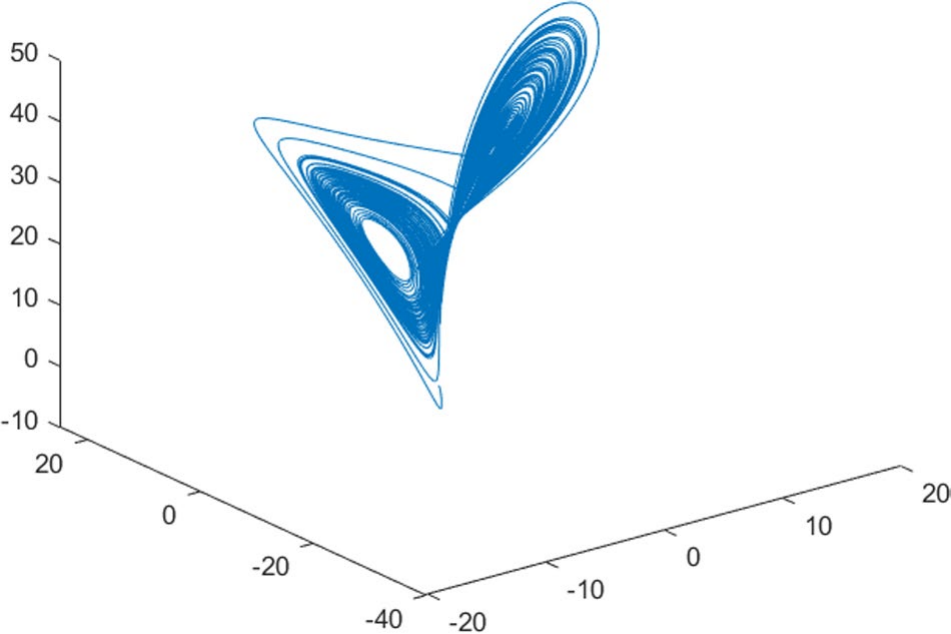
CHA_2 - phase portrait.

**Fig. 5:**
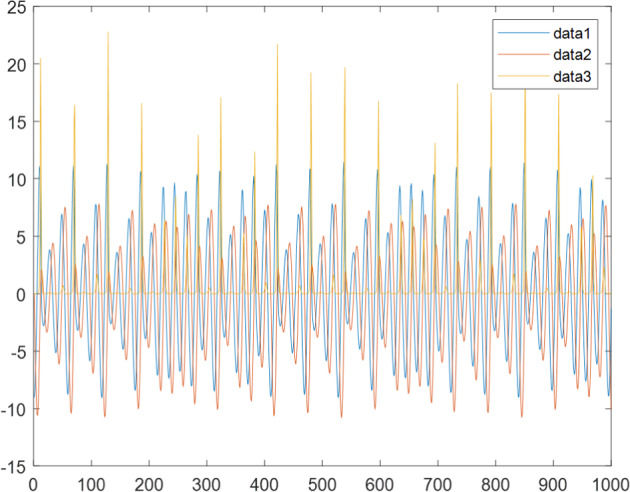
CHA_3 - sample signal.

**Fig. 6:**
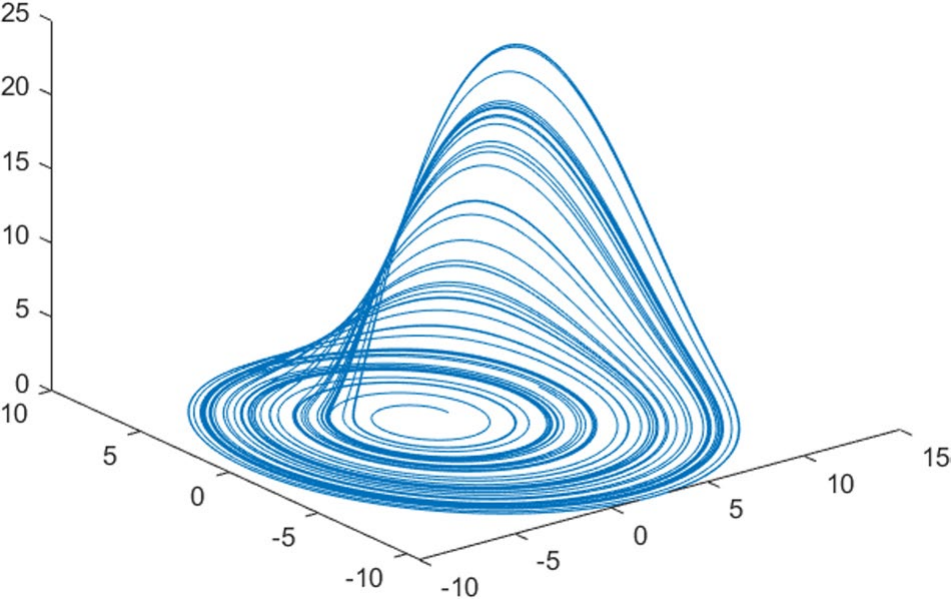
CHA_3 - phase portrait.

**Fig. 7:**
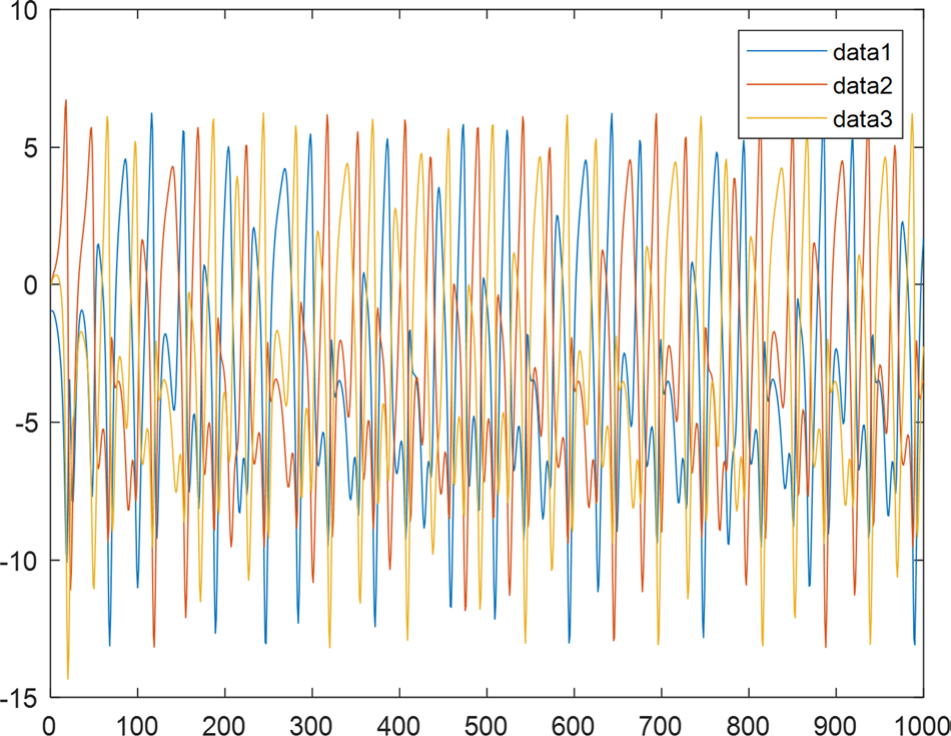
CHA_4 - sample signal.

**Fig. 8:**
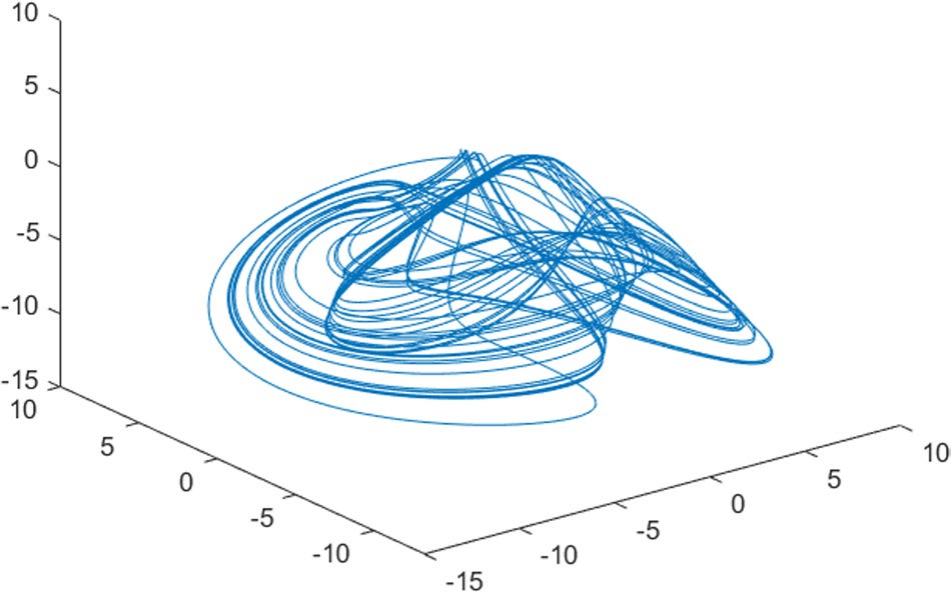
CHA_4 - phase portrait.

**Fig. 9:**
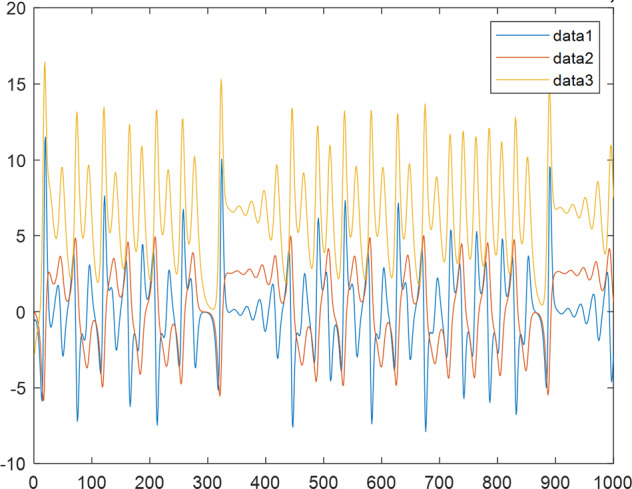
CHA_5 - sample signal.

**Fig. 10:**
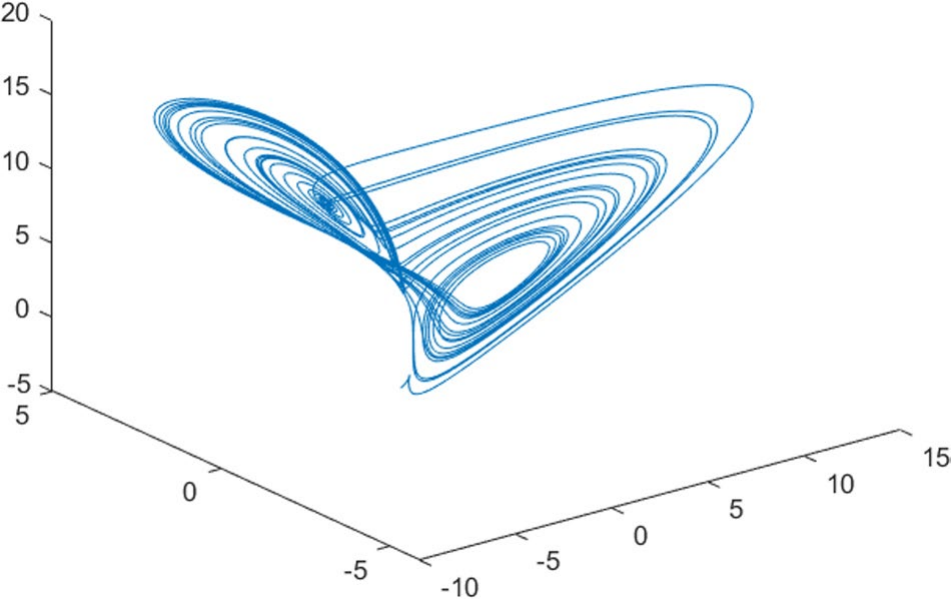
CHA_5 - phase portrait.

**Fig. 11:**
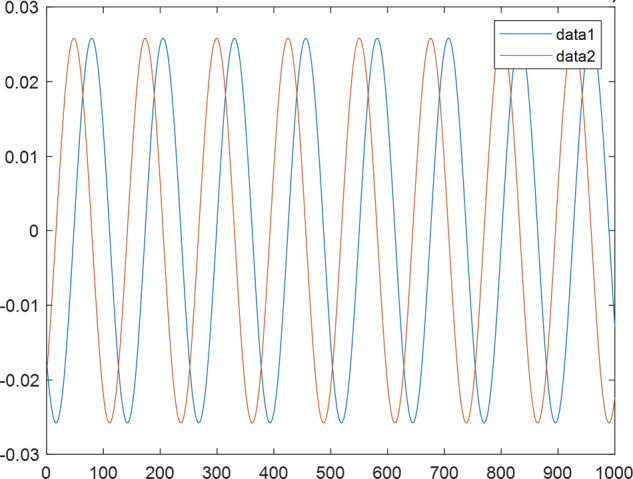
OSC_1 - sample signal.

**Fig. 12:**
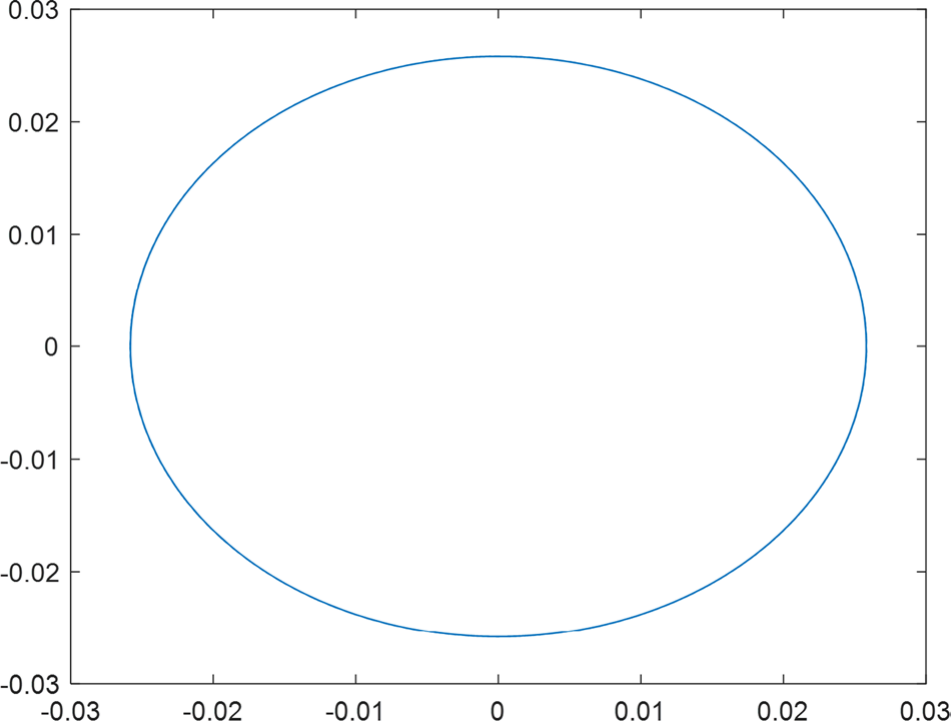
OSC_1 - phase portrait.

**Fig. 13:**
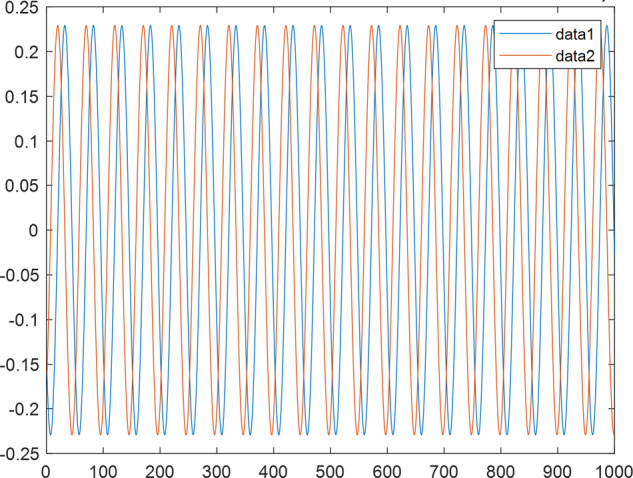
OSC_2 - sample signal.

**Fig. 14:**
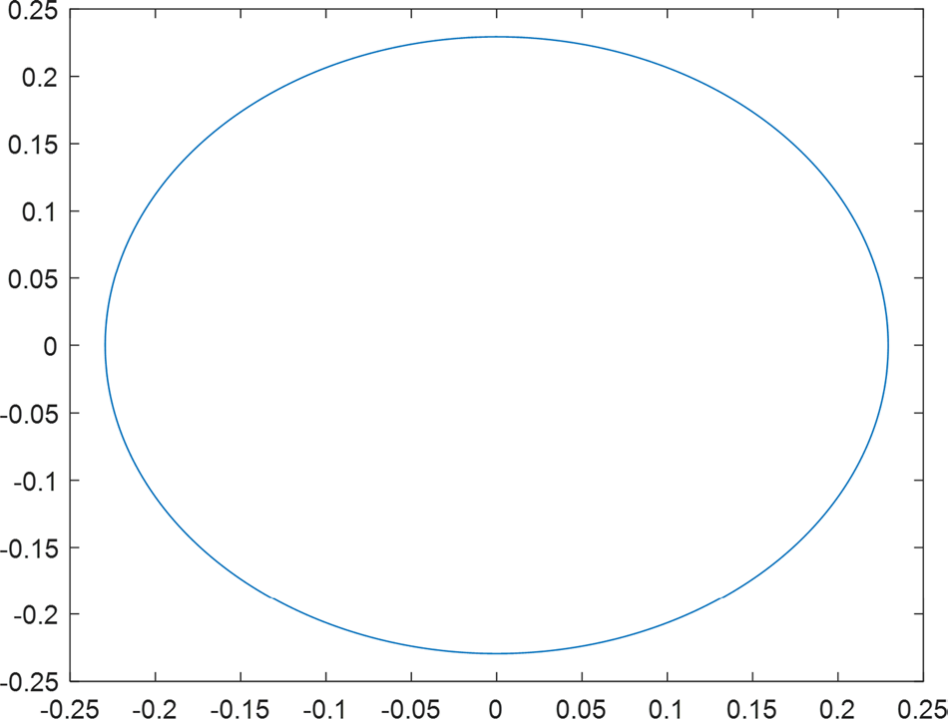
OSC_2 - phase portrait.

**Fig. 15:**
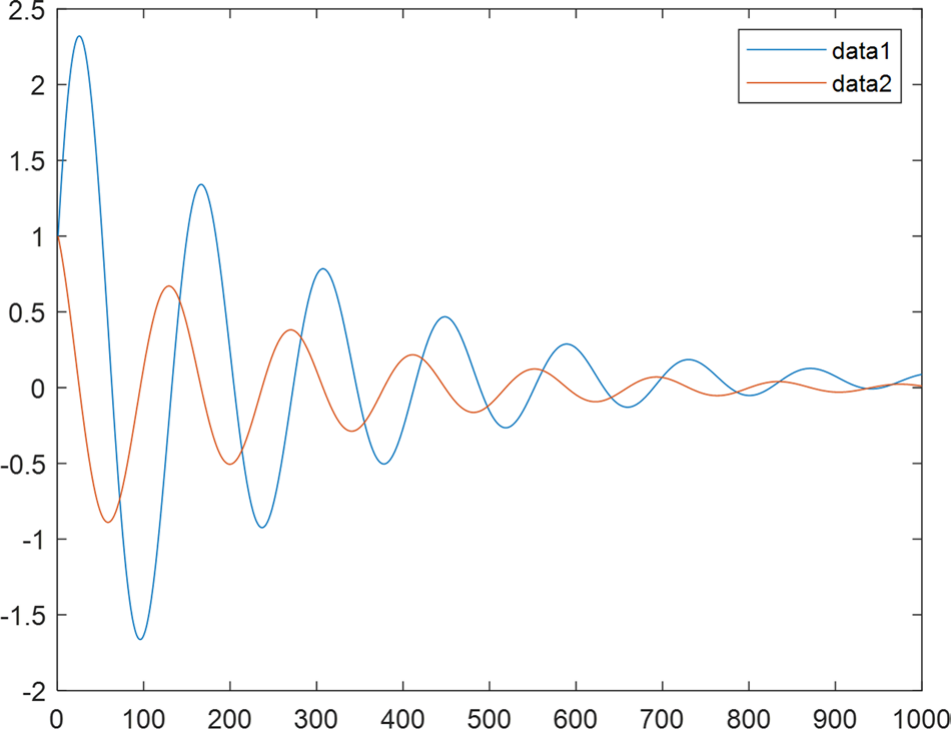
DOSC_1 - sample signal.

**Fig. 16:**
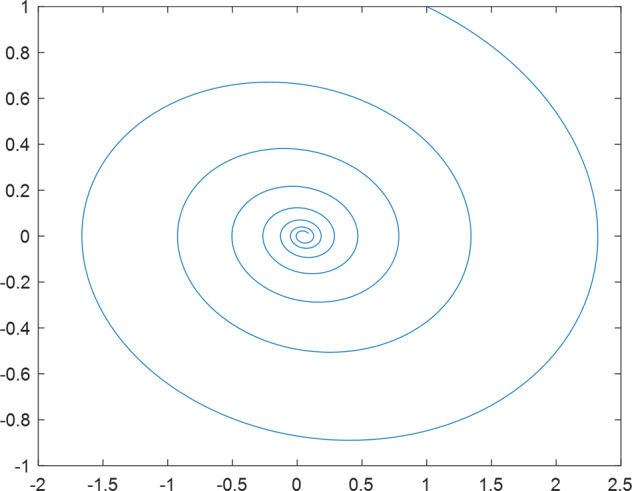
DOSC_1 - phase portrait.

**Fig. 17:**
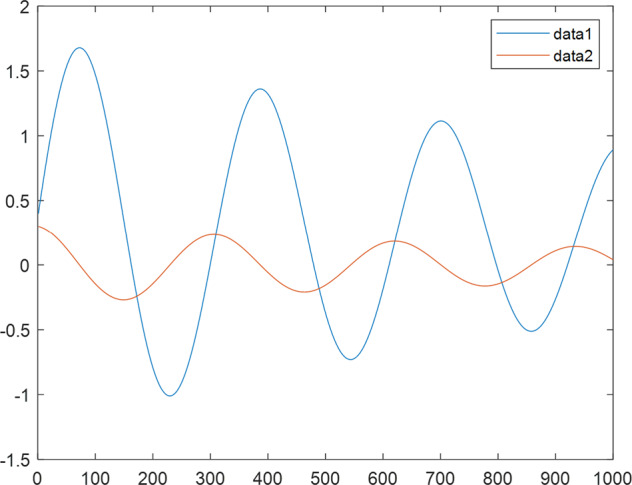
DOSC_2 - sample signal.

**Fig. 18:**
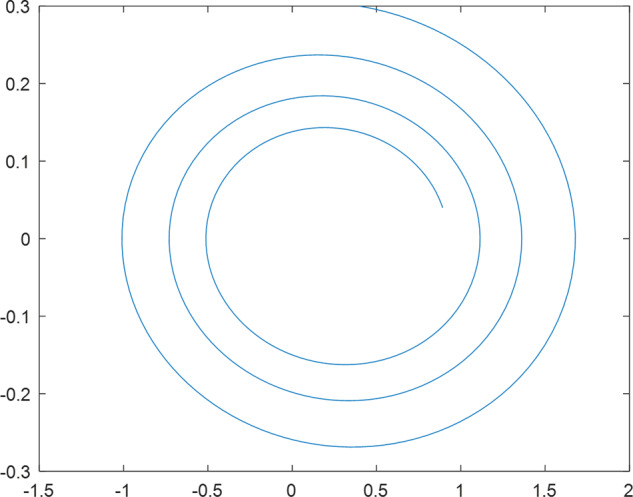
DOSC_2 - phase portrait.

**Fig. 19:**
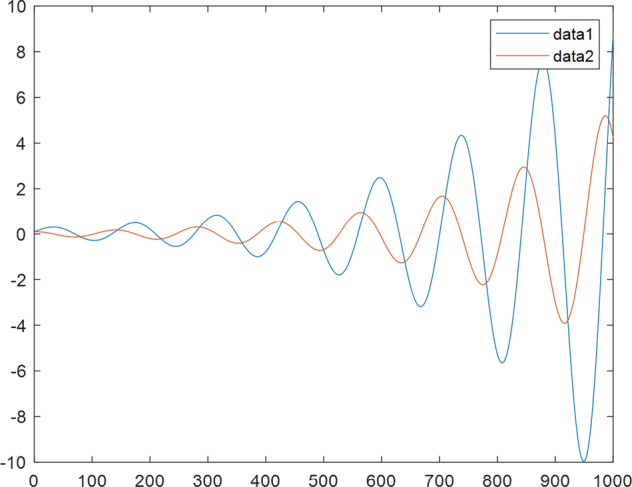
IOSC - sample signal.

**Fig. 20:**
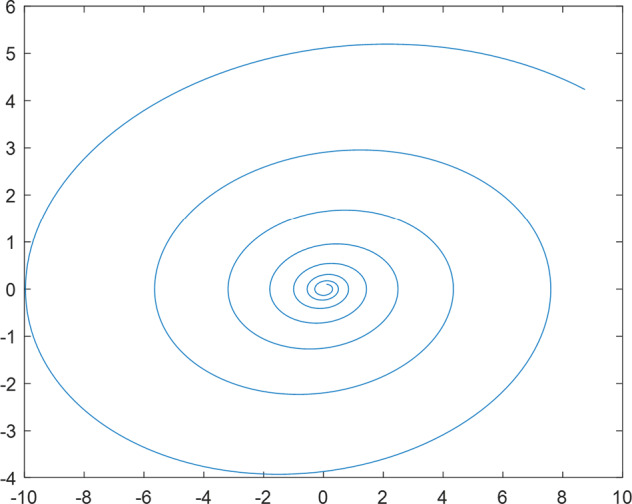
IOSC - phase portrait.

**Fig. 21:**
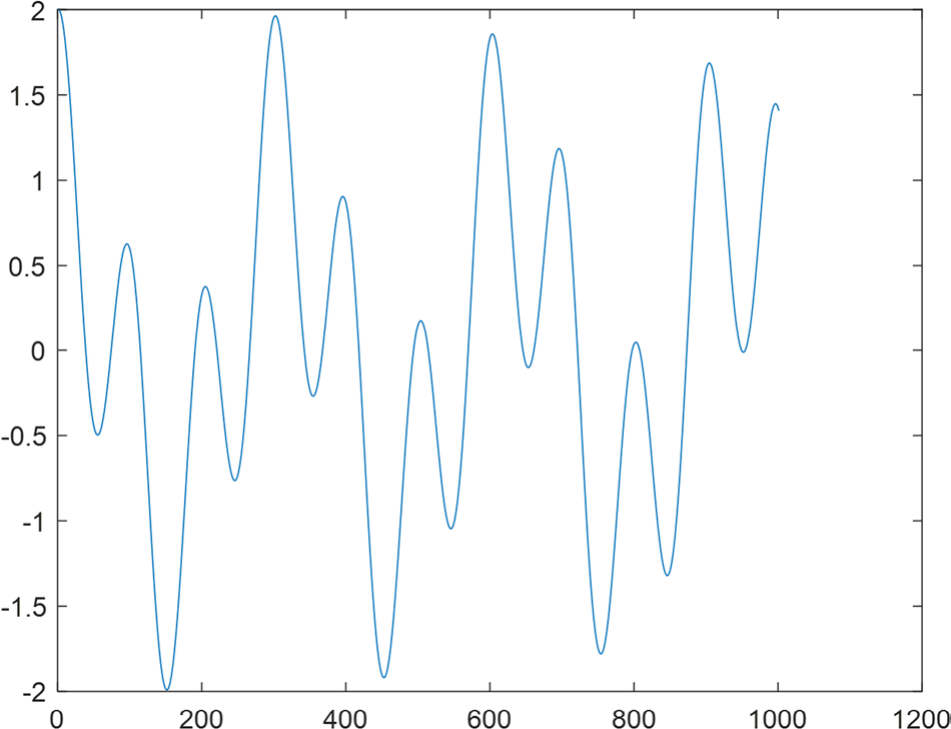
QPS_1 - sample signal.

**Fig. 22:**
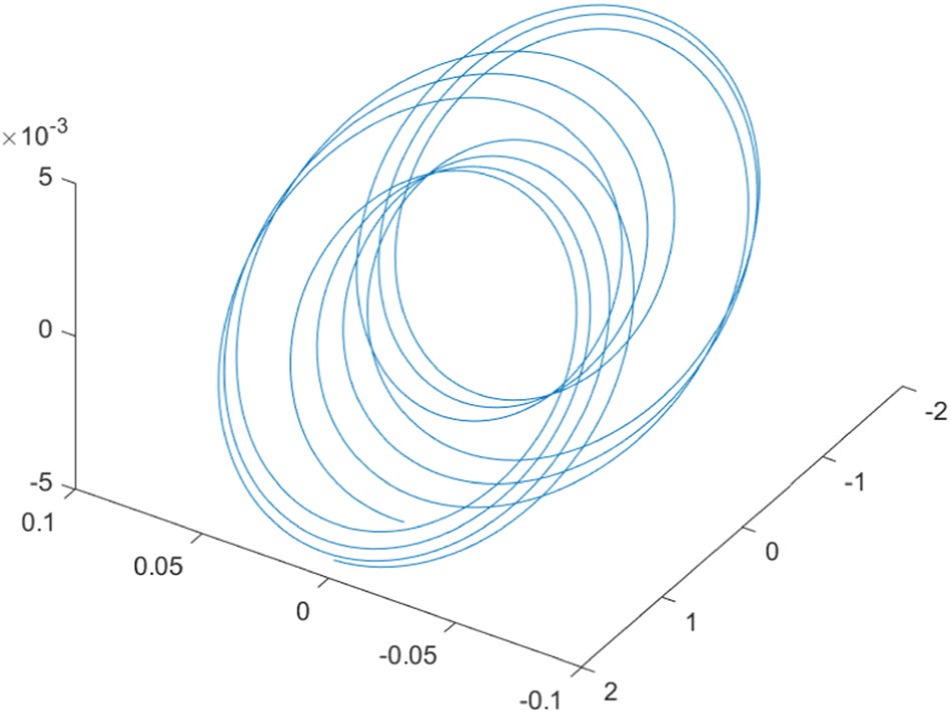
QPS_1 - phase portrait.

**Fig. 23:**
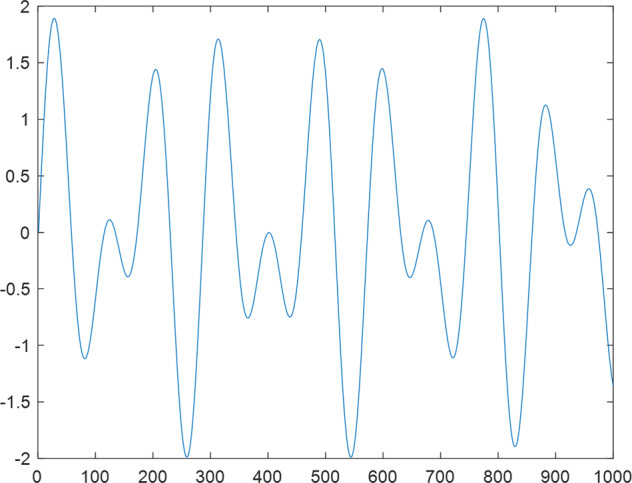
QPS_2 - sample signal.

**Fig. 24:**
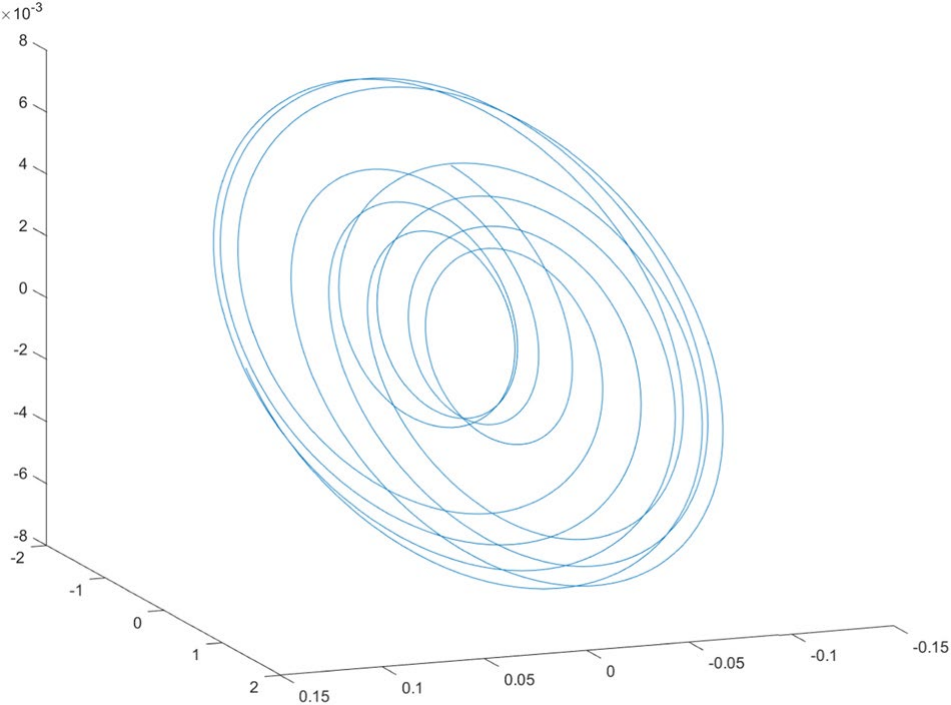
QPS_2 - phase portrait.

**Fig. 25:**
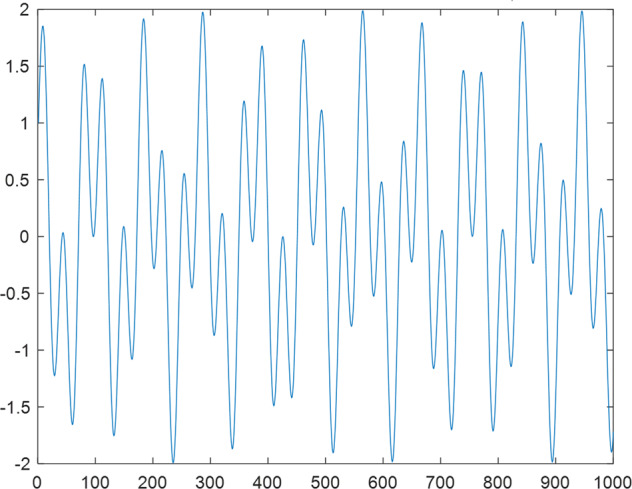
QPS_3 - sample signal.

**Fig. 26:**
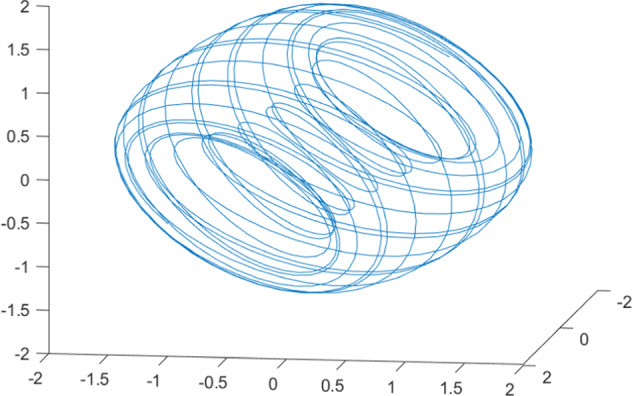
QPS_3 - phase portrait.

**Fig. 27:**
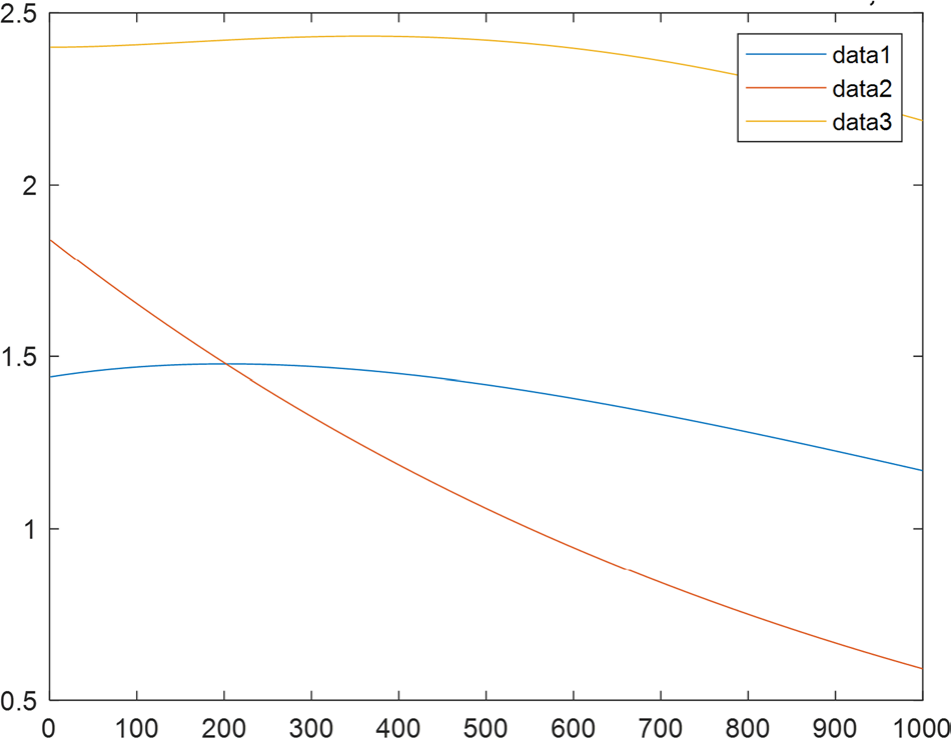
DS_1 - sample signal.

**Fig. 28:**
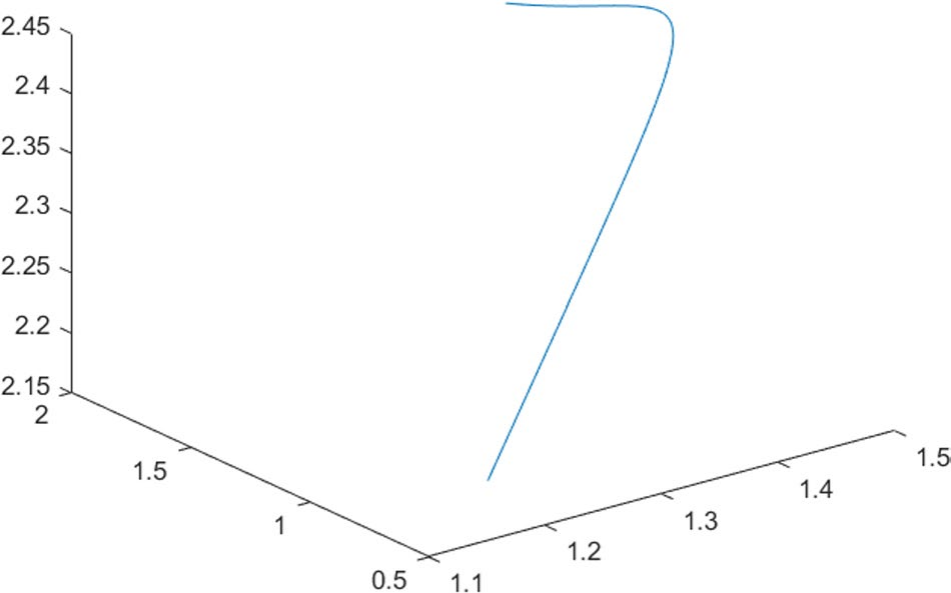
DS_1 - phase portrait.

**Fig. 29:**
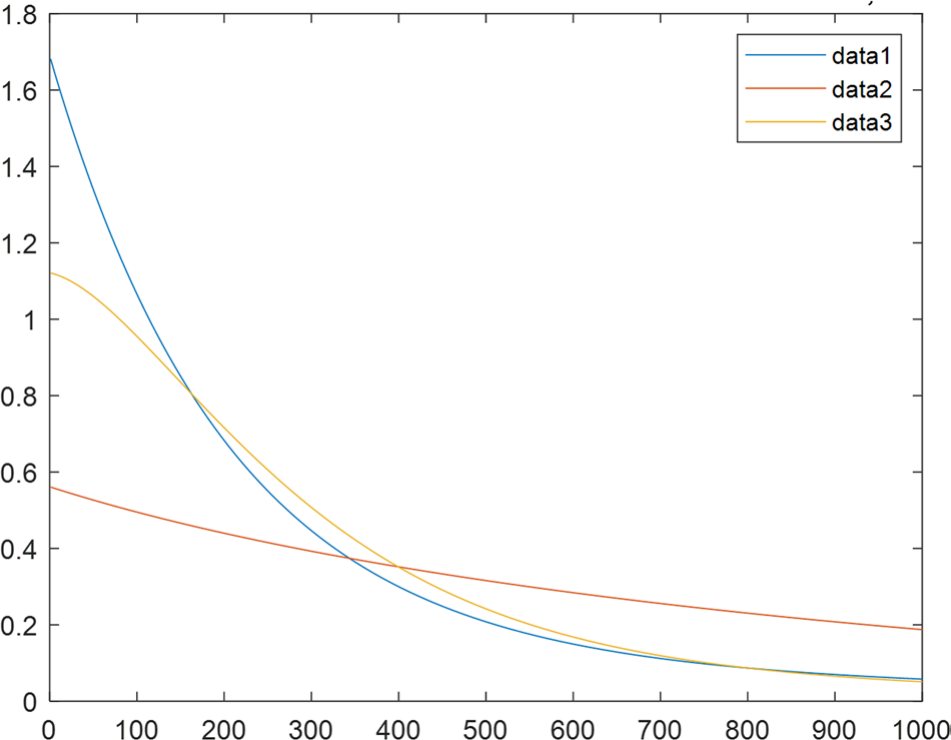
DS_2 - sample signal.

**Fig. 30:**
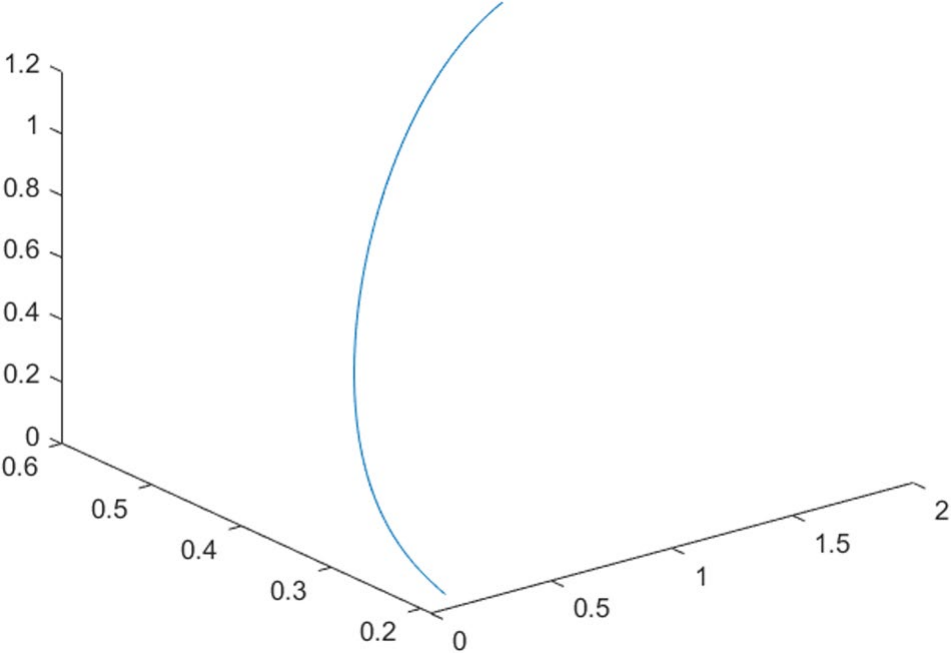
DS_2 - phase portrait.

**Fig. 31:**
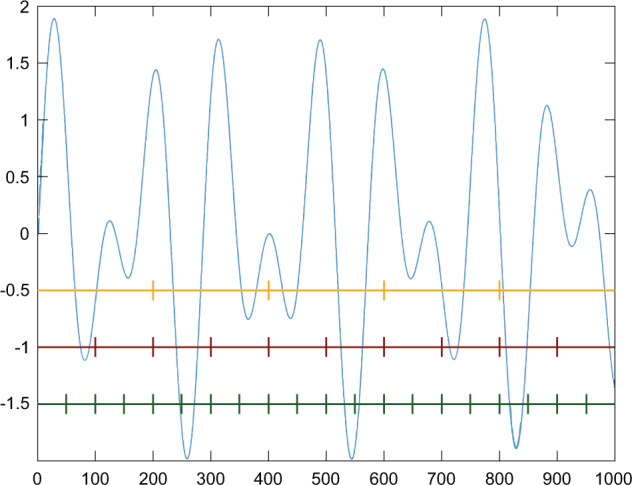
An example signal course with the segments of different sizes marked. Yellow represents 200, red 100, and green 50 element parts. An example of a whole signal course divided into segments.

**Table 5:** The number of samples in the train and test sets. Experiments 1 and 2, when the whole signals were considered.

Segment	Train Set (exp. 1 and 3)	Test Set (exp. 1)	Test Set (exp. 3)
50	340,000	253,040	313,100
100	170,000	126,520	156,550
200	85, 000	63,260	78,275

The training set was additionally divided into two subsets. 75% of samples were used for training a model, while 25% for its validation. The shape of arrays including vectors for training and testing the LSTM network was (X, Y, 1), where X represents the number of samples in a given set (as in Table 5), and Y ∈{50, 100, 200} depending on the segment size used. In the case of the CNN model, the array’s shape was (X, Y, 1, 1).

During the analysis of the proposed dataset usability, the simple LSTM and CNN network models for time series classification were tested. When searching for an efficient model, it turned out that simple architectures were sufficient to obtain satisfactory results.

The recurrent network consisted of four layers: input layer, LSTM layer with four nodes, dropout layer with the dropout rate of 0.5, and dense layer with sigmoid as an activation function (Fig. 33). The convolutional network architecture included a one-dimensional convolution layer (Conv1D) with the activation function calculated by rectified linear unit (RELU), batch normalization layer, dropout layer with the dropout rate of 0.5, and flatten and dense layers (Fig. 34). All models were compiled with the categorical cross-entropy loss function and an Adam optimizer with a learning rate 0.001 was used to train the model. The batch size for fitting the model was set to 64.

**Fig. 32:**
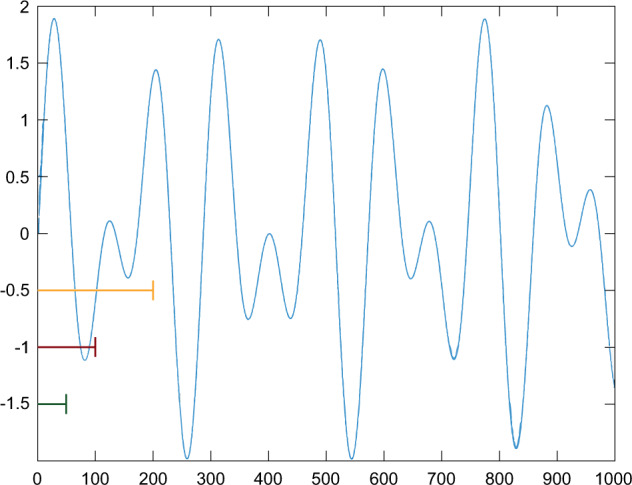
An example signal course with the segments of different sizes marked. Yellow represents 200, red 100, and green 50 element parts. An example of the first segments of a different size from a signal course.

**Fig. 33:**
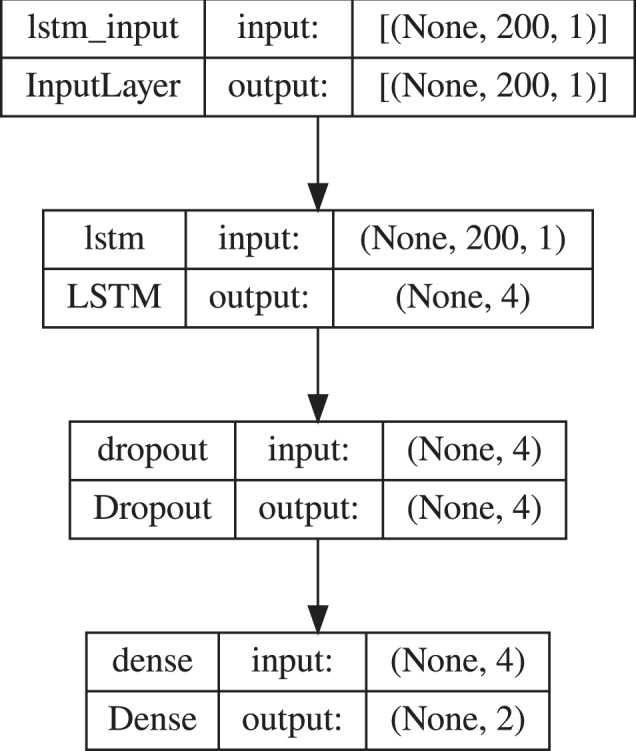
The LSTM model used in the experiment 1.

**Fig. 34:**
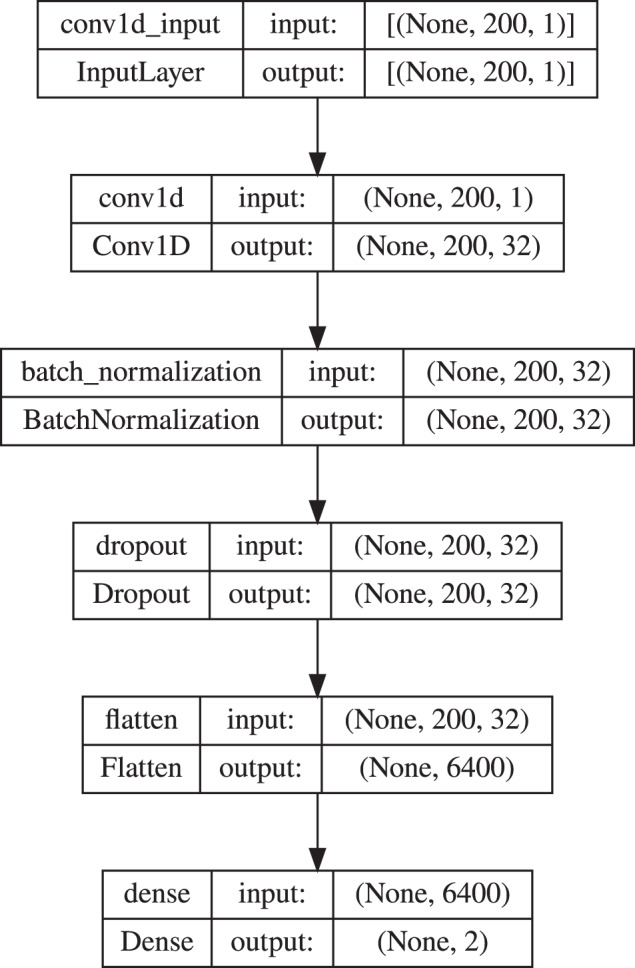
The CNN model used in the experiment 1.

The achieved results presented in Table 6 revealed a very good accuracy for both network types, reaching 95% or higher. Columns from TP to FP show how the models dealt with samples coming from both classes. In this juxtaposition, the signals with chaotic behaviour were denoted as a positive class. It can be noticed that for both models, segments belonging to non-chaotic signals were easier to recognize, whereas chaotic representatives were more often misclassified. Such outcomes for these signals could be expected due to their complex dynamical property. However, when the segment size is considered, similar classification performance was revealed for all course types.

**Table 6:** The results obtained during experiment 1 for various segment sizes and both networks. The chaos class is denoted as Positive and non-chaos as Negative.

Model	Segment	Accuracy	TP	FN	TN	FP
LSTM	50	0.978	107,389	5,651	139,992	8
	100	0.989	55,233	1,287	70,000	0
	200	0.991	27,708	5,52	35,000	0
CNN	50	0.979	108,195	4,845	139,757	243
	100	0.982	54,781	1,713	69,457	543
	200	0.985	27,360	900	34,982	18

### Experiment 2

**Fig. 35:**
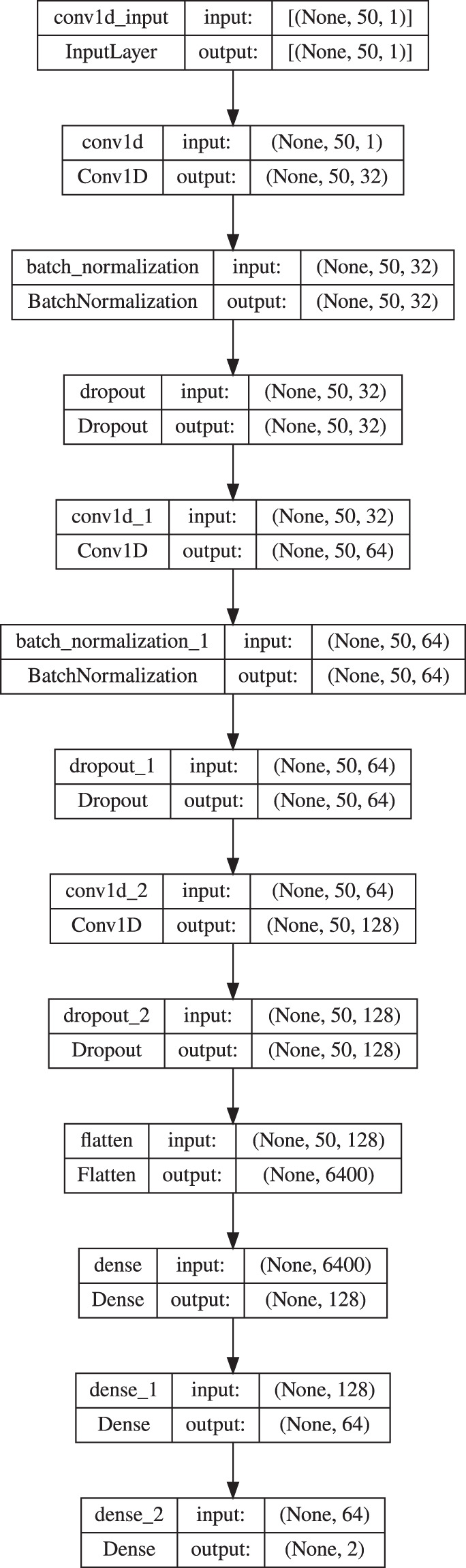
An example CNN model used in experiment 2.

Encouraged by outcomes achieved in the first experiment, the steps toward shortening the training process were taken. The decision was made to reduce the number of samples in the training set. Therefore, to feed the LSTM and CNN networks, only the first segments of each signal were utilized (Fig. 32). Thus, all train and test sets included 17,000 and 12,652 elements, respectively. As can be seen in Table 7 the chosen signal parts proved to be comparably representative as the entire signal in the context of the described classification task realization. However, while the architecture of the LSTM network remained unchanged, the one for CNN had to be extended with more layers to achieve the classification performance on the same level as previously. The example model is visible in Fig. 35. Additionally, LSTM and CNN networks were developed for other segment sizes with input layers consisting of 100 and 200 nodes, respectively.

**Table 7:** The results obtained during experiment 2 for various segment sizes and both networks. The chaos class is denoted as Positive and non-chaos as Negative.

Model	Segment	Accuracy	TP	FN	TN	FP
LSTM	50	0.982	5,485	167	6,942	58
	100	0.977	5,357	295	7,000	0
	200	0.981	5,419	233	7,000	0
CNN	50	0.980	5,650	2	6,744	256
	100	0.979	5,499	153	6,885	115
	200	0.980	5,397	255	7,000	0

### Experiment 3

The subsequent scenario assumed verification of the developed models by the usage of quasi-periodic signals: QPS_1, QPS_2, and QPS_3. Although these signals belong to a non-chaotic class, their characteristics are similar to chaotic ones. Therefore it was expected that the previously prepared models could not be so efficient in determining class labels for these new datasets as in the former experiments. When preparing test sets, quasi-periodic courses were divided into segments according to the previously used patterns. The test set sizes differed for the whole signals’ analysis and are shown in Table 5, column 4. When only the first signals’ parts were taken into account, the test set consisted of 15,652 elements, which is 3,000 more than in experiment 2. The comparison of outcomes achieved in this part of studies shown in Table 8 with those from the previous stages (Table 6) confirms the assumed hypothesis. The same can be noticed when Table 9 is collated with Table 7.

**Table 8:** The results obtained for the whole signals during experiment 3 for various segment sizes and both networks. The chaos class is denoted as Positive and non-chaos as Negative.

Model	Segment	Accuracy	TP	FN	TN	FP
LSTM	50	0.967	107,389	5,651	195,423	4,637
	100	0.985	55,233	1,287	99,022	1,008
	200	0.984	27,708	552	49,352	663
CNN	50	0.924	108,288	4,752	180,873	19,187
	100	0.902	54,781	1,739	86,382	13,648
	200	0.957	27,360	900	47,584	2,431

**Table 9:** The results obtained for the first segment of each signal during experiment 3 for various segment sizes and both networks network. The chaos class is denoted as Positive and non-chaos as Negative.

Model	Segment	Accuracy	TP	FN	TN	FP
LSTM	50	0.937	5,485	167	9,183	817
	100	0.981	5,357	295	10,000	0
	200	0.952	5,419	233	9,488	512
CNN	50	0.951	5,650	2	9,235	765
	100	0.943	5,499	153	9,262	738
	200	0.970	5,397	255	9,785	215

## Usage Notes

First of all, the described datasets can be downloaded from the repository project^34^ or separately for each model (CHA_1^35^, CHA_2^36^, CHA_3^37^, CHA_4^38^, CHA_5^39^, OSC_1^40^, OSC_2^41^, DOSC_1^42^, DOSC_2^43^, IOSC^44^, QPS_1^45^, QPS_2^46^, QPS_3^47^, DS_1^48^, DS_2^49^) and used for training machine learning methods.

Source codes generating described datasets are also available. The published MATLAB programs can be applied to produce new datasets independently. MATLAB source codes of the programs are available in the source-code repository^50^. Sample datasets can be generated by the programs operating in one of the following two modes: for each model separately or for all models. Methods of running the programs are explained in the readme file placed in the mentioned public repository.

## Data Availability

All the shared and previously described datasets were generated by the MATLAB programs. MATLAB Version: 9.9.0.1467703 (R2020b) was used. All MATLAB codes were published through the Gitlab repository^50^. There is no restriction to accessing this public repository of the source code.
